# Multifactorial Optimizations for Directing Endothelial Fate from Stem Cells

**DOI:** 10.1371/journal.pone.0166663

**Published:** 2016-12-01

**Authors:** Drew E. Glaser, William S. Turner, Nicole Madfis, Lian Wong, Jose Zamora, Nicholas White, Samuel Reyes, Andrew B. Burns, Ajay Gopinathan, Kara E. McCloskey

**Affiliations:** 1 School of Engineering, University of California, Merced, United States of America; 2 Graduate Program in Biological Engineering and Small-scale Technologies, University of California, Merced, United States of America; 3 Graduate Program in Quantitative and Systems Biology, University of California, Merced, United States of America; 4 Department of Physics, University of California, Merced, United States of America; 5 Department of Molecular and Cellular Biology, University of California, Merced, United States of America; University of Kansas Medical Center, UNITED STATES

## Abstract

Embryonic stem cells (ESC) and induced pluripotent stem (iPS) cells are attractive in vitro models of vascular development, therapeutic angiogenesis, and tissue engineering. However, distinct ESC and iPS cell lines respond differentially to the same microenvironmental factors. Developing improved/optimized differentiation methodologies tailored/applicable in a number of distinct iPS and ESC lines remains a challenge in the field. Currently published methods for deriving endothelial cells (EC) robustly generate high numbers of endothlelial progenitor cells (EPC) within a week, but their maturation to definitive EC is much more difficult, taking up to 2 months and requiring additional purification. Therefore, we set out to examine combinations/levels of putative EC induction factors—utilizing our stage-specific chemically-defined derivation methodology in 4 ESC lines including: kinetics, cell seeding density, matrix signaling, as well as medium treatment with vascular endothelial growth factor (VEGF), and basic fibroblast growth factor (bFGF). The results indicate that temporal development in both early and late stages is the most significant factor generating the desired cells. The generation of early Flk-1^+^/KDR^+^ vascular progenitor cells (VPC) from pluripotent ESC is directed predominantly by high cell seeding density and matrix signaling from fibronectin, while VEGF supplementation was NOT statistically significant in more than one cell line, especially with fibronectin matrix which sequesters autocrine VEGF production by the differentiating stem cells. Although some groups have shown that the GSK3-kinase inhibitor (CHIR) can facilitate EPC fate, it hindered the generation of KDR+ cells in our preoptimized medium formulations. The methods summarized here significantly increased the production of mature vascular endothelial (VE)-cadherin+ EC, with up to 93% and 57% purity from mouse and human ESC, respectively, before VE-cadherin+ EC purification.

## Introduction

Cell transplantation for therapeutic vasculogenesis is a promising treatment for patients with peripheral vascular disease and severe ischemic heart disease. In studies related to peripheral vascular disease, autologous endothelial progenitor cells (EPC) [1] have been shown to contribute to the formation of collateral arterial vessels and promote the regeneration of ischemic tissues [2–4]. However, it is sometimes difficult to obtain sufficient numbers of proliferating adult EPC, especially from aged and diseased patients [5]. Human embryonic stem cells (ESC) and induced pluripotent stem (iPS) cells, with their unlimited capacity for self-renewal, are considered an excellent potential cell source in a variety of cell-based therapies as well as serve as excellent *in vitro* models of vascular development and tissue engineering.

Endothelial cells (EC) were first successfully derived from both mouse [6–8] and human [9–14] ESC using first three-dimensional (3D) embryoid body (EB) cultures [10, 11, 15] and then 2D cultures with the aid of OP9 cells [12, 13] or mouse embryonic fibroblasts feeder cells [14]. Vascular induction by EB yields very low percentages of EC (1–3%) [10, 11], but EB-monolayer combination inductions [16] and pure monolayer inductions [6, 17–20] lead to greater efficiencies compared with 3D EB differentiation methods. Recently, chemically-defined mediums have been used in feeder-free monolayer cultures for the induction of larger numbers of EC from both mouse [21] and human ESC [9], and allow the development of improved approaches for directed differentiation including a labor intensive method sprouting endothelial progenitor cells (EPC) into 3D fibrin scaffolds [22]. Methods for EC and pericyte co-differentiation have also been developed [23, 24], directing iPS cells in defined medium supplemented with BMP-4 (or Activin), VEGF, and the GSK3-kinase inhibitor (CHIR) generating cultures containing 15–25% CD31^+^/CD34^+^ EPC and up to 50% PDGFRβ mesenchymal cells after 10 days.

The role of small-molecule signaling in vascular differentiation has been getting more attention in recent years. Specifically, the temporal activation of canonical Wnt signaling using a Wnt agonist, GSK3β inhibitor (CHIR-99021), has been shown to promote earlier mesoderm fate [25, 26], while the TGFβ receptor type 1 inhibitor (SB431542) can minimize smooth muscle cell proliferation in differentiating EC cultures [27]. Most recently, the incorporation of seeding density and GSK3-inhibition optimization generated over 50% CD31^+^/CD34^+^ EPC in one line of iPS cells with multipotent aptitude [25], but still required much longer times to generate mature VE-cadherin+ EC.

Literature suggests that the most potent EC inducing biomolecules include: VEGF, bone morphogenic protein-4 (BMP-4), and bFGF. At early stages of commitment, BMP-4 and VEGF promote ventral mesoderm and hematopoietic development while inhibiting neuronal development [28–30], whereas, mitogenic VEGF and bFGF are important at later stages. At low levels, BMP-4 induces mesoderm and subsequent EC differentiation from Flk-1/KDR+ cells [31] through the phosphorylation of the Flk-1 and Tie-2 receptors. The third biochemical, bFGF, in combination with VEGF, is known to promote angiogenesis [28], and upregulate EC markers in EPC [32]. Although a wide array of additional EPC and EC promoting factors have been identified, many of these mimic activation of the same signaling pathways activated by VEGF, BMP-4, and bFGF. [33, 34].

Despite our growing understanding of the critical biochemical factors in development, the precise timing and quantitative levels of EC induction/activation for directing vascular fate from ESC *in vitro* remains confounding. For example, the optimal time to induce mESC-D3 mouse ESC into Flk-1^+^ VPC has been reported to occur at day 4 [6, 35, 36] while the optimal time for the corresponding mESC-R1 induction has been reported at day 2 [17]. VEGF is the most published growth factor associated with directly influencing EC differentiation, but published treatment levels vary between 20ng/ml and 50ng/ml [6, 7, 37]. Matrix signaling is also an important variable in directing stem cell fate, but studies on this topic have also been conflicting. First, it was reported (data not shown) that collagen type-IV directed a greater percentage of EC [6, 7, 37], however; more recent studies show that fibronectin and collage-type IV can generate a comparable percentages of EC. Moreover, fibronectin also promotes increase cell adhesion and/or proliferation of vascular progenitor cells (VPC) [17]. Now, the cell seeding density has been shown to be another important factor in directing EC fate from one line of iPS cells [25]. New studies highlight that both ESC and iPS cells require a step-wise approach to optimizing variables at multiple stages of cardiac commitment for each distinct cell line [38], but this has not yet been examined in EC fate.

**Fig 1:**
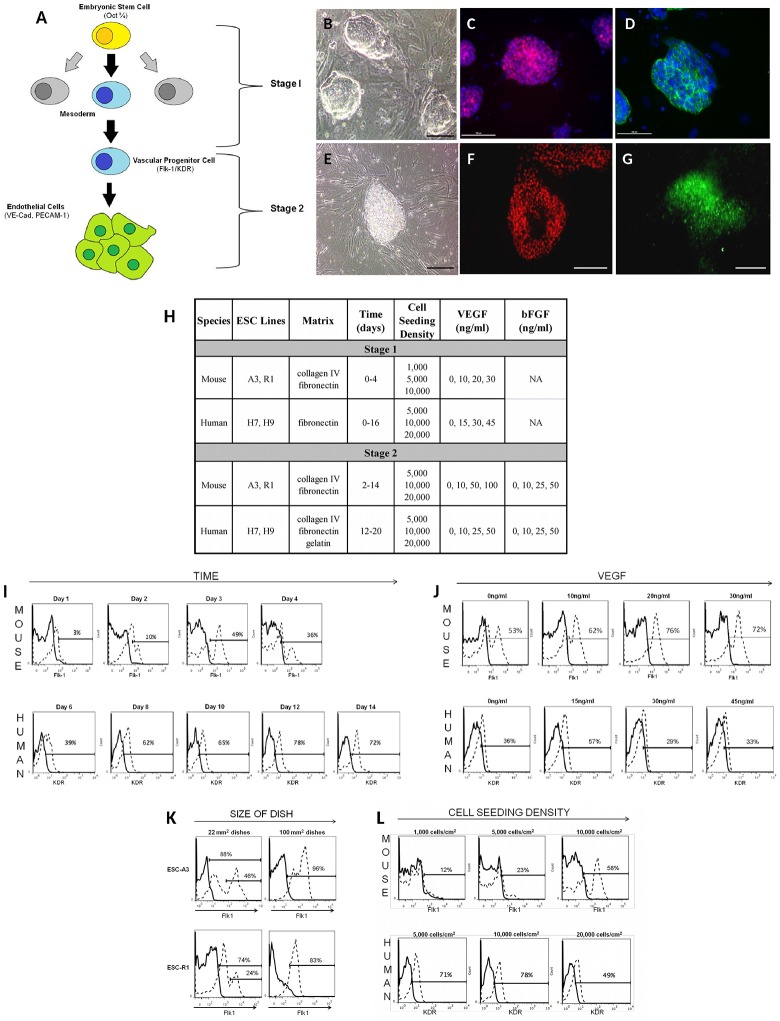
Putative variables directing ESC Differentiation into EC. **A)** ESC first differentiate into mesodermal Flk-1/KDR+ VPC (stage 1), and subsequently commit and mature into EC (stage 2). The Flk-1+ and KDR^+^ markers identify VPC for mouse and human cells, respectively. The subsequent EC are identified by VE-cadherin^+^ expression. **B-G)** The mouse and human ESC lines used exhibit markers consistent with pluripotency. Mouse ESC-A3 exhibited **B)** colony morphology and stained positive for **C)** Oct¾ (red) and **D)** SSEA-1 (green); counterstained for DAPI (blue);scale = 100μm. Human ESC-H7 exhibited **E)** colony morphology and stained positive for **F)** Oct¾ (red) and **G)** SSEA-4 (green); scale = 100μm. **H)** Summary of the ranges of conditions that were analyzed for induction of ESC into Flk-1/KDR+ VPC and VPC into VE-cadherin+ EC. Stage 1 conditions included matrix signaling, kinetics, cell seeding density, and amounts of VEGF supplemented in the chemically-defined cell culture medium. Stage 2 additionally examined the bFGF concentration required in the cell culture medium. Four ESC lines were used in this study: two mouse ESC lines (A3 and R1) and two human ESC lines (H7 and H9). **I)** Representative histograms of the Flk-1+ expression of mESC-A3 and KDR+ expression hESC-H9 as they change over time. These mESC-A3 were induced by seeding cells 10,000 cells/cm^2^ on fibronectin with 20 ng/mL VEGF. The percentage of Flk-1+ from mESC-A3 increased from day 1 to a maximum at day 3. The hESC-H9 were induced by seeding cells at 10,000 cells/cm^2^ induced on fibronectin with 15 ng/mL VEGF with the number of KDR+ cells peaking at day 12. **J)** Representative histograms of the Flk-1+ expression of mESC-A3 and KDR+ expression hESC-H9 at increasing levels of VEGF treatment. For mESC-A3, cells were induced on fibronectin at 10,000 cells/cm and data collected on day 3 of differentiation. The hESC-H9 were also induced on fibronectin at 10,000 cells/cm with the data collected on day 12 of differentiation. **K)** Representative histograms of the Flk-1+ expression of mESC-A3 and KDR+ expression human hESC-H9 as a function of cell seeding density. For mESC-A3, cells were induced on fibronectin with 20ng/ml VEGF and data collected on day 3 of differentiation. The hESC-H9 were induced on fibronectin with 15 ng/ml VEGF and the data was collected on day 12 of differentiation. Flk-1 percentages increase with higher seeding densities, based on a density of 1,000 cells/cm^2^ to a maximum of 10,000 cells/cm^2^. Both mESC and hESC generate the greatest percentage of VPC at cell seeding density of 10,000 cells/cm^2^. **L)** When mESC cells were induced (stage 1) on larger 100mm^2^ plates, Flk-1 expression increased by approximately 10% for both mESC-A3 and mESC-R1 cells.

The presented multifactorial studies examined combinations of up to 5 distinct variables in 2 mouse and 2 human ESC lines using our previously published staged and chemically-defined differentiation methodologies for these studies [17]. We have defined the differentiation of pluripotent ESC into Flk-1^+^/KDR^+^ VPC [7] as “Stage 1” and the specification and maturation of the VPC into vascular endothelial-cadherin positive (VE-cad^+^) EC as “Stage 2” (Fig 1A). The variables examined for early stage 1 induction of VPC included: time, cell seeding density, matrix substrate, and treatment levels of VEGF, while the variables examined for late stage 2 induction included: time, cell seeding density, matrix substrate, and treatment levels of both VEGF and bFGF. Lastly, we examined the putative role of Wnt agonist to aid VPC fate [39, 40]. The results highlight that some factors driving VPC and EC fate are conserved across both mouse and human ESC lines, and some factors vary between species and/or ESC lines of the same species. Most importantly, we show that VEGF treatment is not statistically significant during induction of most lines of ESC.

## Methods

### Mouse ESC induction towards Flk-1+ VPC (stage 1)

The mESC (A3 and R1 lines) were harvested using trypsin/EDTA (Mediatech) and plated with either 50 ng/mL collagen-type IV (BD Biosciences) or 50 ng/mL fibronectin (BD Biosciences) in basal medium: alpha-minimal essential medium (MEM; Cellgro), 20% KSR (Invitrogen), 1Χ penicillin-streptomycin (ps, Invitrogen), 1Χ nonessential amino acids (NEAA; Invitrogen), 2 mM L-glutamine (Invitrogen), 0.05mM 2-mercaptoethanol (Calbiochem), and 5 ng/mL BMP-4 (R&D Systems), supplemented with either 0 ng/mL, 10 ng/mL, 20 ng/mL, or 30 ng/mL of VEGF (R&D Systems) and induced for between 1–4 days. Experiments were repeated in triplicate (N = 3).

### Human ESC induction towards KDR+ VPC (stage 1)

Human ESC (H7 and H9 lines) were dissociated with 1x trypsin/EDTA for 3 minutes, plus pipette physical dissociation into small clumps of 2 to 4 cells. Trypsin was then inhibited with soybean trypsin inhibitor (Gibco), and plated on dishes coated with 10 ng/mL fibronectin at either: 1,000, 5,000, or 10,000 cells/cm^2^. The Stage I induction medium included: alpha-MEM (Cellgro), 20% KSR (Invitrogen), 1Χ ps, 1Χ NEAA, 2 mM L-glutamine, 0.05 mM 2-mercaptoethanol, and 5ng/mL BMP-4 with either: 0 ng/mL, 15 ng/mL, 30 ng/mL, or 45 ng/mL of VEGF and either 0 or 12μM [39, 40] of the GSK3β inhibitor CHIR-99021 (Selleckchem). Cells were examined for expression KDR+ every other day up to day 14 or 16, with a full media change every 3^rd^ day. Experiments were repeated at least twice (N = 2–4).

### Mouse ESC induction towards VE-cadherin+ EC (stage 2)

Mouse ESC-R1 were first sorted for Flk-1^+^ expression using an Aria III Fluorescence Activated Cell Sorter (FACS; BD Biosciences). The scaled-up early stage induction of Flk-1^+^ mESC-A3 was found to be consistently above 90% and did not require sorting. The Flk-1^+^ VPC were plated on 50 ng/ml collagen-type IV (BD Biosciences) or 50ng/ml fibronectin (BD Biosciences) at either: 5,000, 10,000, or 20,000 cells/cm^2^. The stage 2 medium consisted of: 70% alpha-MEM (Mediatech) and 30% DMEM (Invitrogen), 2Χ Nutridoma CS (Roche), 1Χ ps, 1Χ NEAA, 2 mM L-glutamine, and 0.05 mM 2-mercaptoethanol. The supplemental amounts of bFGF (Sigma) examined included: 0 ng/ml, 10 ng/ml, 25 ng/ml, or 50 ng/ml; while amounts of VEGF examined were: 0 ng/ml, 10 ng/ml, 50 ng/ml, or 100 ng/ml. Partial media changes occurred every other day. After 7 days, cells were harvested for analysis.

### Human ESC induction towards VE-cadherin^+^ EC (stage 2)

KDR^+^ VPC induced from stage were first labeled with biotinylated anti-VEGF-2/KDR^+^ ((Miltenyi Biotech) followed by Streptavidin MicroBeads (Miltenyi Biotech), and processed through a MACS magnetic separation column (Miltenyi Biotech) according to the manufacturer recommendations. The KDR^+^ VPC were plated on dishes coated with either: 10 ng/ml fibronectin (BD Biosciences), 50ng/ml collagen-type IV (BD Biosciences), or 0.5% gelatin at either: 5,000, 10,000, or 20,000 cells/cm^2^. The stage 2 medium consisted of: 70% alpha-MEM and 30% DMEM, 2Χ Nutridoma CS, 1Χ ps, 1Χ NEAA, 2 mM L-glutamine, and 0.05 mM 2-mercaptoethanol. The supplemental amounts of bFGF examined included: 0 ng/ml, 10 ng/ml, 25 ng/ml, or 50 ng/ml; while amounts of VEGF examined were: 0 ng/ml, 10 ng/ml, 25 ng/ml, or 50 ng/ml. Half media changes were performed every 3 days. The cells were collected 2 weeks following the stage 1 induction, at days 28 and 26 of total differentiation for H7-ESC and H9-ESC, respectively.

### VE-cadherin expression over time

Mouse ESC-derived VPC were derived under optimal stage 1 and 2 conditions and analyzed on days 5, 7, 10, 12, and 14. Experiments were done in triplicate (N = 3). Human ESC-derived VPC were derived under optimal stage 1 and 2 conditions and collected every 3 days from day 21 through day 53 (N = 3).

### Human iPS cell induction towards KDR^+^ VPC (stage 1)

Human iPS cells were detached by treating 100mm^2^ dishes with 4 mL of TrypLE (Thermo Fisher) for 5 minutes followed by physical dissociation by triturating with a pipette. An aliquot of the cells was counted and plated on 10 μg/mL fibronectin-coated dishes at 1,000, 5,000, or 10,000 cells/cm^2^. The Stage 1 mesoderm induction media consisted of alpha-MEM, 20% KSR, 1x ps, 1x NEAA, 2 mM L-glutamine, 0.05 mM 2-mercaptoethanol, and 5 ng/mL BMP-4 with either 15 ng/mL or 30 ng/mL of and either 0 or 12μM [39, 40] of the GSK3β inhibitor CHIR-99021. Cells were cultured for 10 days before collection, with a full media change every third day.

### Low density lipoprotein uptake of ESC-derived EC

EC were seeded at 40,000 cells/cm^2^ in plates coated with 50 ng/mL (mESC) or 10 ng/mL fibronectin (hESC) for the mESC-A3 and hESC-H7, respectively. Once confluent (about 3 days) the cells were incubated with Alexa Fluor 488 acetylated low density lipoprotein (LDL; Invitrogen) diluted 1:100 in DMEM with high glucose (Invitrogen) for 5 hours. The wells were then counterstained with DAPI and fixed with 4% paraformaldehyde.

### Matrigel-based tube formation assay

Matrigel^™^ (BD Bioscience) was added to several wells—500μl each—and allowed to solidify for 30–60 minutes at room temperature. EC derived from mouse ESC-A3 and human ESC-H7 were added to individual wells at 40,000 cells per well in 500 μL. Cells were imaged after 24 and 48 hours. Transmission images were captured using either a Nikon Eclipse TE2000-U fluorescence microscope with NIS-Elements AR 3.1 Software or a Fisher Scientific^™^ Micromaster^™^ Infinity Optics Microscopes with Micron Software.

### ESC induction towards Flk-1^+^ VPC using insoluble VEGF

ESC were induced towards Flk-1^+^ VPC under three different conditions. VEGF was provided as either soluble VEGF supplemented in the chemically defined medium, insoluble VEGF mixed with fibronectin (FN:VEGF), or both. ESC were seeded at 10,000 cells/cm^2^ and cultured in induction medium, previously optimized by our laboratory [17], consisting of alpha-MEM, 20% KSR, 1× ps, 1×NEAA, 2 mM l-glutamine, 0.05 mM 2-mercaptoethanol, 20 ng/mL of VEGF, and 5 ng/mL BMP-4. The expression of VEGR receptors Flk-1 and Flt-1 were examined at the time in which that ESC line generated greatest numbers of Flk-1^+^ VPC—2 and 3 days for the R1-ESC [17] and A3-ESC [41], respectively.

### Flow cytometry analysis

Adherent cells were harvested using Cell Dissociation Buffer (Invitrogen) or TrypLE (iPS cells) fixed in 4% paraformaldehyde (Tousimis), rinsed 2Χ with PBS, blocked using 0.5% donkey serum (Fitzgerald) and 1% bovine-serum albumin (Sigma) for 1 hour at room temperature (RT), and permeabilized (if needed) with 0.7% Tritron X-100 (MP Biomedicals) in PBS. For Stage 1, cells were stained with either 1:100 Alexa Fluor 647^®^-conjugated anti-Flk-1 antibody (Biolegend) or 1:50 Flk-1 rabbit polyclonal antibody (Santa Cruz Biotechnology) followed by 1:100 secondary donkey anti-rabbit FITC (Biolegend) or 1:75 anti-human CD309/VEGFR2 PE monoclonal (Biolegend) and BD Biosciences 1:1000 human Fc Block (1:1000) followed by 1:75 mouse IgG1, κ°PE (Biolegend) was used for the isotype control. For Flt-1 expression on mouse VPC, cells were stained with goat IgG anti-Flt-1 (Santa Cruz) or goat IgG (Abcam) for one hour followed by anti-goat FITC (Santa Cruz) at 1:200. Stage 2, cells were stained with CD144 (VE-cadherin) PerCP-efluor710^®^ at 1:200. All cells were counterstained with 1:1000 eFluor 780 Fixable Viability Dye (eBioscience) and analyzed on an LSR II Flow Cytometer System (BD Biosciences).

### Immunofluorescence

Mouse ESC-R1 (mESC-R1) and hESC-H7 (hESC-H7) derived EC were grown to confluence and fixed with 4% paraformaldehyde. Cells were permeabilized with 0.7% Triton X-100 and blocked with 5% donkey serum (Fitzgerald) and 1% Bovine Serum Albumin (Sigma) prior to staining against goat VE-cadherin (Santa Cruz) and rabbit PECAM-1 (Santa Cruz) followed by anti-goat TRITC and anti-rabbit FITC (Santa Cruz), Alexa Fluor-488^®^ anti-Phalloidin (Invitrogen) or Alexa Fluor-567^®^ anti-Phalloidin (Invitrogen), and DAPI.

### Modeling and statistical analysis

As is standard for many experimental methods in the field, initial studies included three replications (N = 3) of each combination of conditions. However, it quickly became apparent that the exorbitant cost of growth factors and serum replacements required to conduct all combinations of conditions in triplicate in all 4 cell lines was going to be unaffordable. Therefore, subsequent studies were conducted at N = 1, with subsequent regression analysis performed over the continuous range of levels. Unfortunately, N = 1 limited our ability to conduct statistical analysis of the discrete matrices examined in the study even though the impact of matrix signaling was often robust. To identify the importance of the continuous variables (time (day), cell seeding density, and growth factor treatments) to the outcomes (Flk-1/KDR+ VPC and VE-cadherin^+^ EC), we performed multiple regression analyses using both linear and non-linear models. We used the MATLAB Statistical Toolbox’s **fitlm** function to estimate the linear model coefficients that best fit our data. The linear models that we used to fit the data for stage 1 and stage 2 are:

% VPC = a*day + b*density + c*VEGF + k (1)

% EC = a*bFGF + b*density + c*VEGF + k (2)

where ***a***, ***b***, and ***c***, and ***k*** are model parameters and their best fit values (S1A & S1B Fig) were independently confirmed using LASSO with 10-fold cross-validation (MATLAB Statistical Toolbox’s **lasso** function) to fit our data. Coefficients with p-values less than 0.05 were considered significant.

To estimate optimum induction conditions that could generate the greatest number of Flk-1/KDR^+^ VPC and VE-cad^+^ EC, we utilized a non-linear regression model containing all possible linear and quadratic terms. For example, for three dependent variables x_1_, x_2_, x_3_ (e.g. bFGF, seeding density and VEGF respectively for stage 2), the fitting function used was

$$\%\;EC\;=\;b_{1}\;+\;b_{2}*{\text{x}}_{1}\;+\;b_{3}*{\text{x}}_{2}\;+\;b_{4}*{\text{x}}_{3}\;+\;b_{5}*{\text{x}}_{1}{}^{2}\;+\;b_{6}*{\text{x}}_{2}{}^{2}\;+\;b_{7}*{\text{x}}_{3}{}^{2}\;+\;b_{8}{*\text{x}}_{1}{*\text{x}}_{2}\;+\;b_{9}{*\text{x}}_{2}{*\text{x}}_{3}\;+\;b_{10}{*\text{x}}_{1}{*\text{x}}_{3}$$ (3)

Using the MATLAB Statistical Toolbox’s **fitnlm** function, we estimated the best fit values of these model parameters. Finally, by maximizing the fitted function within the range of the variables experimentally accessible, we were able to predict optimal conditions.

## Results

The quality of undifferentiated mESC and hESC were assessed for expression of pluripotency markers: Oct ¾ and SSEA-1 (mouse) or SSEA-4 (human), and colony morphology (Fig 1B–1G). Putative signals were examined more rigorously in all combinations for their ability to direct Flk-1/KDR^+^ VPC and VE-cad+ EC. Early stage (stage 1, Fig 1H) induction factors included: kinetics of differentiation (i.e. time, Fig 1J), matrix signaling, cell seeding density (Fig 1J), and level of VEGF treatment (Fig 1K), and late stage (stage 2) induction factors included: kinetics of differentiation, matrix signaling, cell seeding density and both bFGF and VEGF levels of treatment (stage 2, Fig 1H). Each combination of conditions was examined and compared against all other combination of conditions. The experiments were then reproduced in two mouse and two human ESC lines, mESC-A3 and mESC-R1 and hESC-H7 and hESC-H9, respectively. The large sets of biological data were analyzed for statistical significance using multiple linear regression models.

### Induction towards Flk-1/KDR^+^ VPC (stage 1)

**Fig 2:**
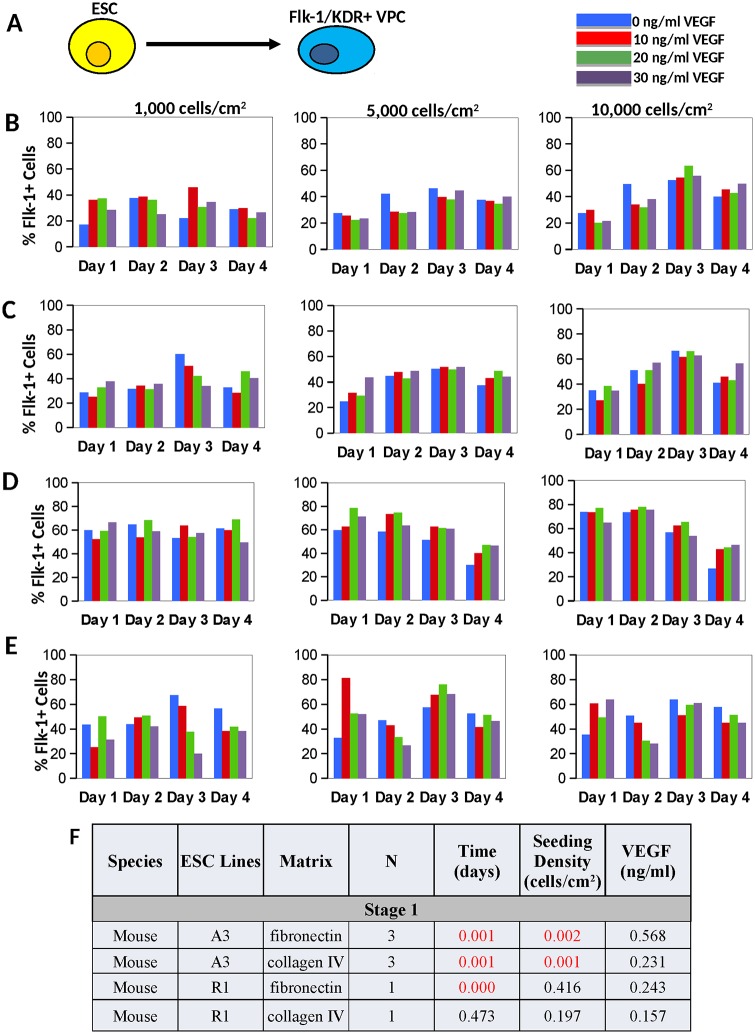
Results from stage 1 mouse ESC inductions. **A)** ESC were induced into Flk-1/KDR+ VPC. Bar graphs show results from differentiation of mESC-A3 into Flk-1+ VPC on **B)** fibronectin (N = 3) and **C)** collagen IV (N = 3) over time and for a range of cell seeding densities and VEGF treatment levels. Optimal induction conditions were achieved on day 3 at the greatest cell seeding density. Bar graph shows results of differentiating mESC-R1 into Flk-1+ VPC on **D)** fibronectin (N = 1) and **E)** collagen IV (N = 1). Optimal induction conditions for this cell line peaked between days 1–2 on fibronectin. **F)** Based on regression analyses, P-values are provided for stage 1 mESC induction variables, with statistical significance (p < 0.05, highlighted in red). Note that VEGF treatment was not found to be statistically significant at any level.

**Fig 3:**
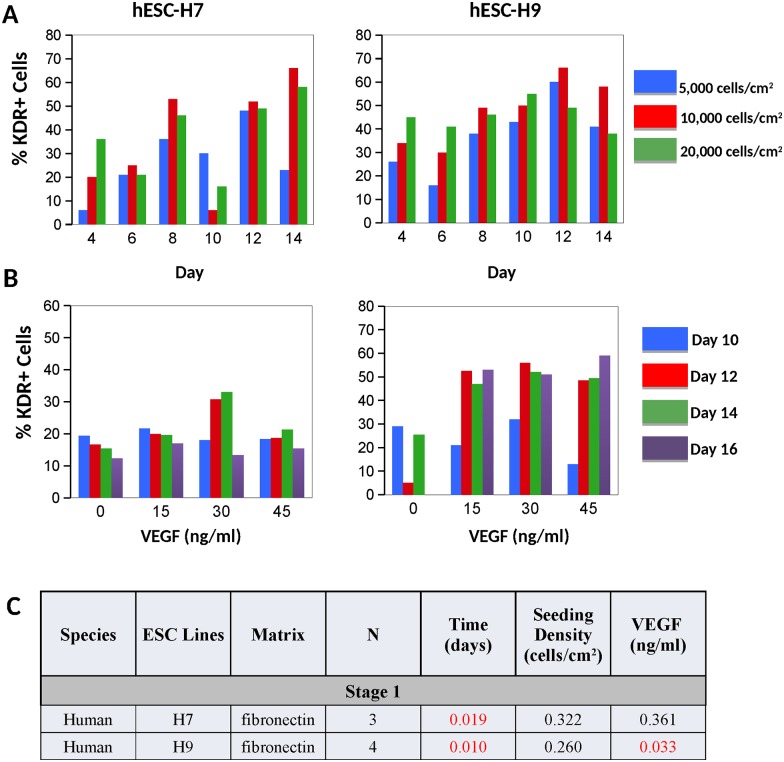
Results from stage 1 human ESC inductions. Induction of **A)** hESC-H7 and hESC-H9 were examined for peak expression of KDR+ VPC over time and a range of cell seeding densities (N = 1). The best differentiation conditions were achieved on days 14 and 12 for hESC-H7 and hESC-H9, respectively. **B)** The human ESC were then induced for range of VEGF treatments (N = 3). **C)** Based on regression analyses, P-values are provided for stage 1 hESC inductions, with statistical significance (p < 0.05, highlighted in red). Here VEGF was significant in the hESC-H9 line only.

While fibronectin and collagen-type IV generated approximately equivalent numbers of VPC from mESC-A3 (Fig 2A & 2B), for mESC-R1 fibronectin matrix generated greater percentages of VPC compared with collagen-type IV (Fig 2C & 2D). Fibronectin also generated the greatest number of VPC from both human ESC lines (not shown). For all ESC lines, the induction of Flk-1/KDR^+^ VPC is temporally regulated, with the greatest levels of VPC expressed within a short time window, and then subsequently receding (Figs 2 and 3). This temporal regulation is distinct in mouse and human ESC lines. The percentage of Flk-1/KDR^+^ VPC was the greatest between days 2–3 in mouse ESC (Fig 2B–2F) and between days 12–14 in human ESC (Fig 3). Specifically, the Flk-1^+^ cell expression peaked at day 2 in mESC-R1 and day 3 in mESC-A3, while the KDR^+^ expression in hESC-H7 and hESC-H9 peaked at days 14 and 12, respectively. Multiple regression analyses confirmed that time is the most significant factor (p < 0.05) in the differentiation of Flk-1/KDR^+^ cells in both mouse and human ESC (Figs 2F and 3C, respectively).

Optimal cell seeding density was conserved at 10,000 cells/cm^2^ in both mouse and human ESC (Figs 2B–2F and 3), and a robust factor in directing VPC in the mESC-A3 line (p-value = 0.001 on collagen and p-value = 0.002 on fibronectin, Fig 3C).

The VEGF treatment level, although considered to be a key signal in directing VPC fate in many studies [7, 8, 42], was not found to be an important nor a statistically significant factor in either mouse ESC line (Fig 2F). The KDR^+^ induction from hESC-H9 is the only cell line that exhibited a statistically significant increase in VPC above 0 ng/ml of VEGF treatment (Fig 3). Moreover, the optimal level of VEGF treatment for VPC commitment varies from cell line-to-cell line (Figs 2 and 3), suggesting that the optimal VEGF signaling may be dependent on properties inherently distinct in each ESC line.

### Scaling up

In order to generate enough cells for the late stage experiments, the plate size was increased from 12-well dishes to 100 mm^2^ plates. In scaling up for late stage differentiation, it was noted that the larger plate size led to greater percentages of Flk-1^+^ cells, increasing from 65–67% up to 80–95%, in both the A3 and R1 ESC lines (Fig 1L).

### Induction of VPC in to VE-cadherin+ EC (stage 2)

All ESC lines were first differentiated into VPC (stage 1) using their optimized conditions and purified, if necessary, based on Flk-1/KDR^+^ expression. For stage 2 induction, the purified mESC or hESC were examined on fibronectin, collagen, or gelatin over a range of cell seeding densities, VEGF and bFGF treatments. Data was collected from mESC after an additional week of culture following optimal stage 1 induction, at day 9 total for mESC-A3 and day 10 total for and mESC-R1. The cells from hESC inductions were collected 2 weeks following the stage 1 induction, at days 28 and 26 of total differentiation for H7-ESC and H9-ESC, respectively.

**Fig 4:**
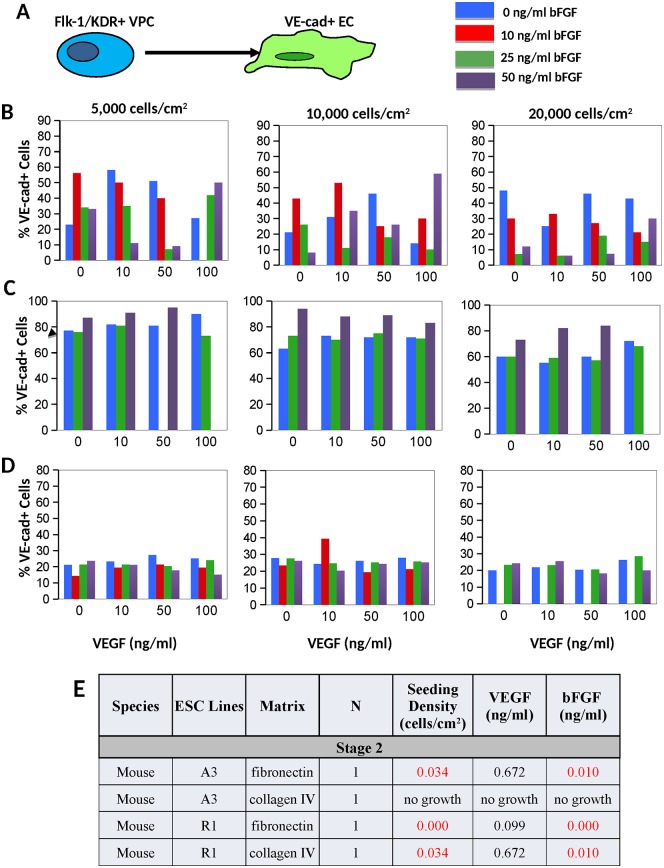
Results from stage 2 inductions of mouse ESC. **A)** VPC were induced into VE-cad+ EC. **B)** Results of differentiating mESC-A3 VPC on fibronectin (N = 1). The A3-ESC Flk-1+ VPC failed to adhere to the collagen type-IV substrate, so no data is available for that condition. Results of differentiating mESC-R1 VPC on **C)** fibronectin (N = 1), and **D)** collagen IV (N = 1). **E)** Based on regression analyses, P-values are provided for stage 2 mESC inductions, with statistical significance (p < 0.05, highlighted in red). The seeding density and bFGF treatment level were both found to be statistically significant variables, but not VEGF treatment levels.

**Fig 5:**
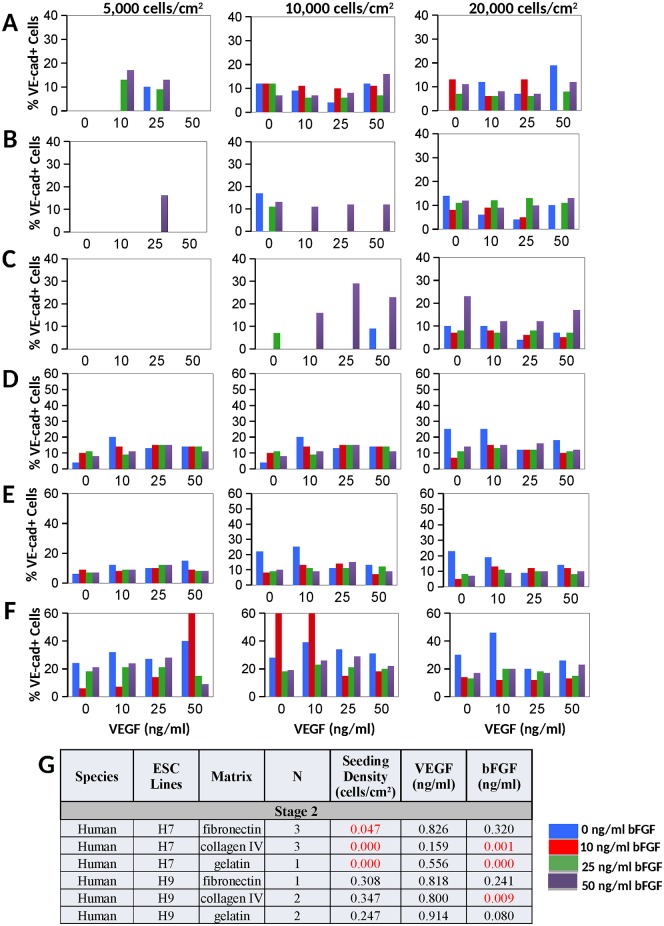
Results from stage 2 induction of human ESC. Bar graphs show results from inductions of hESC-H7 VPC into VE-cadherin+ EC on **A)** fibronectin, (N = 1), **B)** collagen IV (N = 1), and **C)** gelatin (N = 1); and inductions of hESC-H7 VPC into VE-cadherin+ EC on **D)** fibronectin, (N = 1), **E)** collagen IV (N = 1), and **F)** gelatin (N = 1). **G)** Based on regression analyses, P-values are provided for stage 2 hESC inductions, with statistical significance (p < 0.05, highlighted in red). The seeding density was significant using the hESC-H7 line, but not in the hESC-H9. The bFGF treatment level was sometimes found to be statistically significant, depending on conditions. The VEGF treatment levels were not significant for any of the stage 2 hESC inductions.

The mESC-A3 VPC generated the greatest percentage of VE-cadherin^+^ cells (Fig 4B) at a seeding density of 5,000 cells/cm^2^ (p-value = 0.034) on fibronectin and with medium containing either low or high levels of biochemical treatment: either 10 ng/mL VEGF and 0ng/ml bFGF, or very high levels: 100 ng/ml VEGF and 50ng/ml bFGF (Fig 4B), with statistically significance in bFGF treatment only (p-value = 0.001). Due to poor mESC-A3 adhesion on collagen-type IV following transfer to stage 2, no data was obtained. The mESC-R1 VPC generated up to 95% VE-cadherin^+^ cells when plated on fibronectin at a seeding density of 10,000 cells/cm^2^ (p-value = 0.0001) and treated with a highest, 50 ng/ml, level of bFGF (Fig 4C, p-value = 0.0001) compared with only 21% VE-cadherin^+^ cells when VPC were plated on collagen-type IV (Fig 4D). In the H9-ESC, the greatest numbers of VE-cadherin^+^ EC were generated on gelatin (Fig 5F).

As in early stage inductions, VEGF treatment was not a significant factor in directing any of the cell lines into VE-cadherin^+^ EC. Again, the bFGF treatment exhibited varied and significant signaling in both of the mESC-A3 VPC and mESC-R1 mouse lines (Figs 4E and 5G). Specifically, the A3-ESC VPC generated the greatest percentage of VE-cadherin^+^ EC on fibronectin at 0–10 ng/mL bFGF, while the mESC-R1 cells required 50 ng/mL of bFGF (Fig 4B & 4C). In human ESC lines, the biochemical signaling varied significantly depending on substrate. The VEGF signaling is, again, not a significant factor in the stage 2 induction, while bFGF treatment was significant and distinct between the two cell lines and substrates (Figs 4E and 5G).

The cell seeding density for late stage inductions of mouse ESC lines exhibited significance between 5,000–10,000 cells/cm^2^ (Fig 4E), while the H7-ESC preferred to be seeded at 20,000 cells/cm^2^ (depending on substrate, p-value = 0.000–0.047, Fig 5G). The hESC-H9 seeding on gelatin indicated that a seeding density of 10,000 cells/cm^2^ might be superior, but was not statistically significant.

### Model-based optima

**Fig 6:**
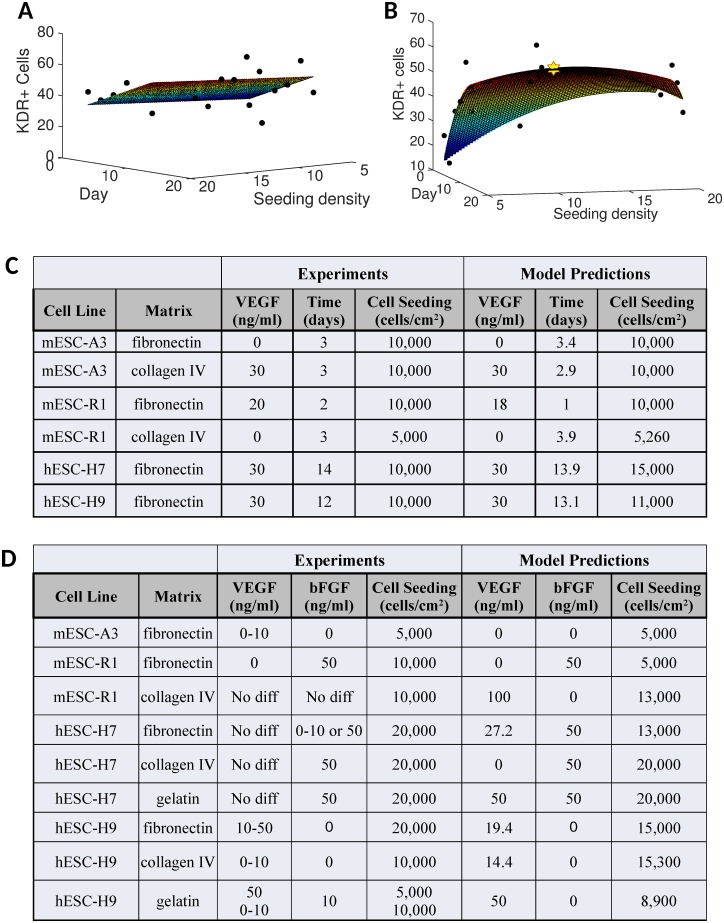
Regression analysis and optimal conditions. Multiple regression analyses using both linear and non-linear models was performed. MATLAB Statistical Toolbox’s **fitlm** function was then used to estimate the linear model coefficients that best fit our data. **A)** A representative 3D graph depicts the best fit linear regression model (surface) and the experimental data (black circles) for hESC-H9 on fibronectin at fixed VEGF but varying time and cell seeding densities. **B)** Representative graph with the same data, but fitted with a non-linear model to identify optimal values (yellow star) of significant variables that maximize KDR+ expression. Experimental optima are presented along with model predictions for **C)** stage 1 inductions of ESC into VPC and **D)** stage 2 inductions of VPC into EC.

The conditions generating optimal Flk-1/KDR^+^ VPC and then VE-cadherin^+^ EC were analyzed using a number of multiple regression models. As described in this section above, we used linear models (see e.g. Fig 6A) that best fit the data to identify the statistically significant variables that control desired expression (as reported in Figs 2–5**)**.

To estimate optimum induction conditions that could generate the greatest number of Flk-1/KDR^+^ VPC and VE-cad^+^ EC, we utilized a non-linear regression model containing both linear and quadratic terms to estimate best fit model parameters. We then maximized the fitted function (e.g. the surface in Fig 6B) within the range of the experimental variables to obtain predictions for the optimal conditions (e.g. the star in Fig 6B). A comparison between the model-based predictions for the optimal conditions and the experimentally estimated optima (Fig 6C and 6D) indicate excellent agreement for both stages. Note that that model can also predict values between measured data points, and therefore, is not limited to the confines of the chosen treatment levels.

### VE-cadherin expression continues to increase over time

**Fig 7:**
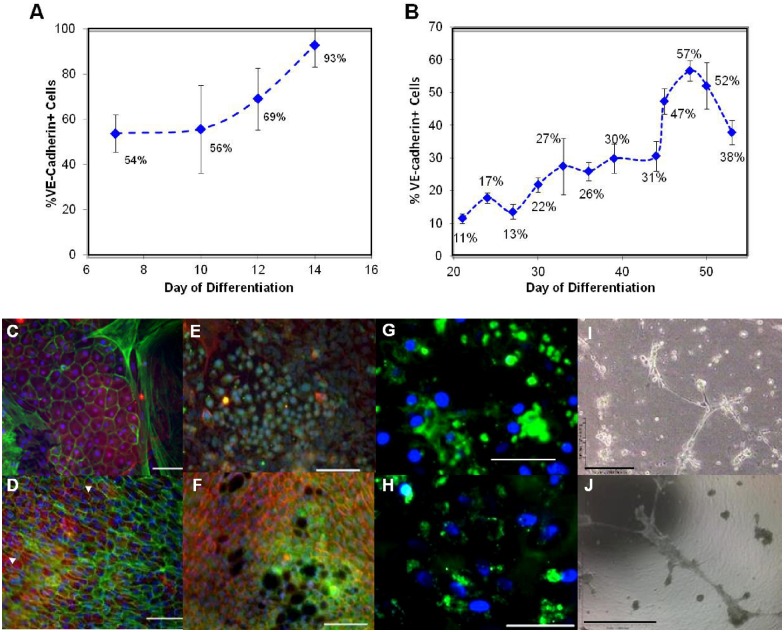
EC Maturation Over Time. After stage 2 induction of Flk-1/KDR+ VPC into VE-cad+ EC, the cells were cultured for longer periods in order to examine further maturation and/or self-purification. Averaged percentages of VE-cadherin+ for both mouse **A)** and human cells **B)** are given. **A)** The mESC-A3 were expanded on fibronectin at 10,000 cells/cm^2^ with 10 ng/mL VEGF and 10 ng/mL bFGF. The mESC-A3 grew to a purity of 93% VE-cadherin by day 14. **B)** The hESC-H7 were cultured on fibronectin, seeded at a density of 10,000 cells/cm^2^ with 25 ng/mL VEGF and 50 ng/mL bFGF. The hESC-H7 also increased in expression of VE-cadherin+ cells, peaking at a 57% by day 48 of total induction (14 days in stage 1 followed by 34 days in stage 2). **C, G, E, I)** Unpurified mESC-R1 derived EC **D, F, H, J)** hESC-H7 derived EC express markers and morphology consistent with EC phenotype: **C-D)** VE-cadherin (red) counterstained with phalloidin (green) and **E-F)** PECAM-1 (green) counterstained with phalloidin (red) Nuclei stained with DAPI (blue). Large mesenchymal-like cells are seen surrounding the murine cells in **C**). The mESC-A3 and hESC-H7 also **(G-H)** took up acetylated LDL and **(I-J)** formed vascular-like structures in Matrigel^™^ within 24 hours.

In order to evaluate the long-term cultures, we examined the percentage of VE-cadherin^+^ cells in these passaged cultures for another few weeks. Representative data from mESC-A3 and hESC-H7 cell lines and are shown (Fig 7A & 7B). For the mESC-A3 cell line, the averaged maximum VE-cadherin purity doubled after 14 days of total differentiation. For the less efficiently induced hESC-H7 cell line, the VE-cadherin almost tripled after 48 total days of differentiation, up to 57%. However, without further purification the human ESC-derived EC the cultures were not able to maintain a high level of purity over longer periods. This is consistent with previous work by our lab and others showing that contaminating fibroblasts and smooth muscle cells can grow and eventually take over EC cultures [43], and may even mitigate EC proliferation [6]. EC selection is required for continued expansion of purified EC.

### Characterization of ESC-derived EC

Final assessment of endothelial of ESC-derived EC was examined using endothelial markers VE-cadherin, PECAM-1, ability to take up low density lipoprotein (LDL) and form vascular-like networks on Matrigel^™^. The stained mESC-R1 and hESC-H7 EC both express VE-cadherin (Fig 7C & 7D) and PECAM-1 (Fig 7E & 7F) diffusely across the cell membrane as well as colocalized at the cell-cell junctions (Fig 7C–7F). The mESC-R1 [44], mESC-A3, and hESC-H7 EC were also able to take up LDL (Fig 7G & 7H), as well as, generate vascular formations on Matrigel^™^ (Fig 7I & 7J). As previously shown by our laboratory and others, the vascular-like networks on Matrigel^™^ would be expected begin to regress after 48 hours [21, 45], but remain viable longer in animal models [46] and 3D in fibrin or collagen gels [47].

### Modulation of Wnt signaling in EC fate

During the course of these studies, the use of iPS cells became more common and the temporal modulation of canonical Wnt signaling, specifically providing early GSK3β inhibition, was shown to increase the generation of CD34^+^/CD31^+^ endothelial progenitor cells from 1% up to 20% [25]. Therefore, we set out to examine the whether the activation of canonical Wnt signaling is beneficial in our optimized hESC cultures. First, we pre-optimized the cell seeding density, VEGF treatment, and induction time for the greatest generation of KDR^+^ VPC from iPS cells (S2A Fig). The iPS cells were then induced with and without the GSK3β inhibitor (CHIR-99021). Using our preoptimized chemically-defined medium, the Wnt agonist did not enhance the generation of KDR^+^ VPC at any time point during the stage 1 induction of either iPS cells or H9-ESC (S2B and S2C Fig, respectively).

### Insoluble VEGF bound in FN

**Fig 8:**
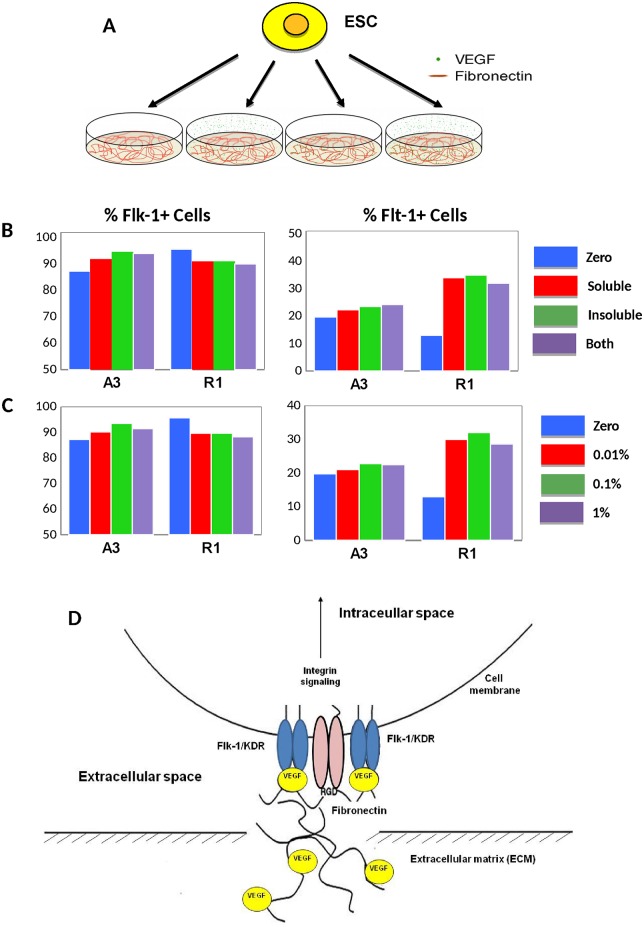
VEGF Presentation. **A)** Experimental schematic for testing the effect of soluble or insoluble VEGF on the differentiation of ESC into vascular progenitor cells. Murine ESC lines R1 or A3 are disassociated into single cells and passed onto fibronectin-coated plates with either zero, soluble VEGF, insoluble VEGF, or both forms of VEGF present. **B)** The presentation of soluble versus insoluble VEGF does not significantly affect the expression of Flk-1 or Flt-1 in either the R1 or A3 mESC cell lines (N = 3, p > 0.05). **C)** Moreover, changing the amount of bound VEGF-FN (either 0%, 0.01%, 0.1% or 1% of occupied VEGF binding sites) does not alter the number of Flk-1+ VPC or Flt-1+ cells (N = 3, p > 0.05). D) Schematic diagram showing VEGF/FN complexes with VEGF binding domains on FN promote integration of signals generated by Flk-1 and α_5_β_1_ (adapted from [48]).

The extracellular matrix (ECM) protein, fibronectin (FN), has been shown to contain two novel VEGF binding domains using direct physical association between the VEGF receptor-2 (Flk-1) and the FN integrin, alpha5beta1 [48] to activate cell signaling cascades FN, thus directing EC fate by sequestering VEGF in an insoluble form [49, 50]. When mixed directly with the FN and incubated overnight, VEGF can also be visualized from day 0–3 (not shown) and an ELISA assay found no detectable VEGF released into the aspirate during the course of the 3-day period (not shown). The effect of providing VEGF as insoluble form, soluble form, or both to mouse R1 and A3 ESC lines was investigated. After 2 or 3 days for the R1-ESC [17] and A3-ESC [41], respectively, the cellular expression of Flk-1 and Flt-1 was quantified for the 4 experimental conditions (Fig 8). The data (N = 3) show that the number of Flk-1^+^ and Flt-1^+^ cells was not significantly altered across all VEGF presentation conditions of soluble and insoluble VEGF supplementation (Fig 8). Although not statistically significant, the expression of decoy VEGF receptor, Flt-1, is mitigated without the additional VEGF supplementation (Fig 8C), especially in the R1-ESC line.

## Discussion

The optimized induction conditions reproducibly generate Flk-1/KDR^+^ VPC (Fig 6C) followed by VE-cadherin^+^ EC (Fig 6D) for the mouse and ESC lines examined with up to 93% efficiency. The inherent kinetics of differentiation was the most important variable in generating high numbers of VPC for both mouse and human ESC. Moreover, the time for mouse ESC differentiation into VPC and EC required much shorter periods compared with human ESC. Specifically, the induction of mouse ESC into VPC required 2–4 days compared to 12–14 days in human ESC. The subsequent VE-cadherin+ EC required 14 total days in mouse compared with 47 total days in human. These independently optimized cell fate kinetics closely mimic the vascular development in the developing embryo: normally occurring between days 10.5–11.5 in the mouse aorta-gonad-mesonephros (AGM) embryonic°regions and days 27–40 in the human AGM [51]–with slightly earlier and later time points in the yolk sac and the fetal liver, respectively. The differentiation of EC from ESC is clearly distinct within species and maintained in the artificial microenvironment of the cell culture dish.

A high cell seeding density was also found to be an important variable in the mouse ESC in generating high numbers of Flk-1^+^ VPC. Moreover, some cell lines seeded at higher densities did not require VEGF supplementation (Figs 2B, 2C and 5F). Because hypoxic conditions are also known to drive mesoderm commitment [52] [53] and endothelial fate [54] from ESC, the molar fraction of oxygen at the cell surface was calculated. Using experimental cell proliferation rates, estimated oxygen solubility in saline solution, and oxygen consumption rates reported for both ESC = 27.5x10^-18^ [55] and EC = 50x10^-18^ mol/cell/s [56], it was determined that, although oxygen would be reduced at higher cell seeding densities, none of the conditions would generate a hypoxic environment defined as 1–3% oxygen. However, it was confirmed that the greater number of Flk-1+ cells in the larger 100-mm dishes (Fig 1L) were receiving almost 2x less media per cell compared to the 12-well plates. Either the nutrient limited environment and/or increased cell-to-cell communication is playing a profound role in EC fate.

Perhaps one of the most important results from this study is that the VEGF treatment was not generally a statistically significant variable in directing EC fate—at either stage of differentiation (Figs 2, 3, 4, 5 and 8), specifically when cells were culture on fibronectin matrix. Note that the presumptive requirement of VEGF treatment in chemically-defined mediums for EC fate has been most rigorously examined using single cells cultured in collagen-type IV coated 96-well plates [7, 8, 57]. This result was initially confusing given the large body of data implicating VEGF signaling in mesoderm and EC fate [7, 8, 37, 58, 59], however, the results provided in this manuscript examining 4 ESC lines indicate that BMP-4 signaling on fibronectin are adequate signals for population-based EPC and EC inductions. We expect that the two distinct VEGF binding domains within FN matrix [48] is sequestering the autocrine VEGF produced by the cells (Fig 8D) and may even be protecting the VEGF from degradation [49, 50]. Our results advocate that the utilization of FN matrix is superior to collagen IV and usually mitigates the need for VEGF supplementation in both VPC and EC induction cultures. Moreover because the BMP in early inductions can also activate the VEGF/VEGFR signaling [34], and bFGF in late induction mediums can activate the same downstream signals as VEGF [60] including the Src family tyrosine kinases, phosphoinositide 3-kinase (PI3K), and p38 MAPK [61] (reviewed in [62]), VEGF treatment is in these medium formulations is excessive and probably an unwarranted expense.

## Conclusions

The combinatorial variables explored and summarized in this manuscript provide information on general and cell line-specific signals that lead to significantly increased the transduction efficiencies of not just EPC, but definitive VE-cadherin+ EC. Optimized protocols generate up to 93% EC from mouse ESC and 57% EC from human ESC before further purification. As reported for cardiac differentiation [38], we show that individual mouse and human pluripotent stem cell lines require some cell-line specific optimization for efficient differentiation. Moreover, a staged approach enables one to tailor the microenvironment signals at early and late phases, corresponding to mesoderm and EC commitment, respectively. The results confirm that the in vitro differentiation kinetics mimic the temporal development in the embryo, and that this is conserved across species. In addition to time, the generation of Flk-1^+^/KDR^+^ VPC from pluripotent ESC is directed predominantly by high cell seeding density and fibronectin matrix signaling, with minimal dependence on VEGF treatment, especially at higher cell seeding densities. The bFGF treatments were found to be the most variable between cell lines.

## Supporting Information

S1 Methods Supplemental Methods for basic mouse and human ESC culture, as well as, generation and characterization of insoluble VEGF in FN. (DOCX)

S1 Fig **Constants from multiple linear regression models** for induction of **A)** ESC into VPC and **B)** VPC into VE-cad+ EC. Example of **C)** trace plots of coefficients fit by lasso and **D)** cross-validated MSE of lasso fits.
(TIFF)

S2 Fig Induction of iPS cells into VPC (stage 1). Human iPS cells were examined for **A)** optimal induction time, seeding density, and VEGF treatment for stage 1 in an iPS cell line. The greatest percentage of KDR+ cells is obtained at day 8 from cells seeding at 1,000 cells/cm^2^, with VEGF treatment between 15 and 30 ng/ml insignificant. The incorporation of a Wnt agonist (GSK3β inhibitor, called CHIRR99021) was examined from day 0–4 in **B)** human iPS cells and **C)** H9-ESC, but did not lead to an increase in KDR+ cells at any time point.
(TIFF)
