# Supplementary material for: Multifactorial Optimizations for Directing Endothelial Fate from Stem Cells
Source: PLoS One. 2016 Dec 1;11(12):e0166663. doi: 10.1371/journal.pone.0166663 (PMC5131944; doi:10.1371/journal.pone.0166663)
Supplement: S1 Methods — (DOCX) [file pone.0166663.s001.docx]

**S1 Methods**

**Mouse ESC Culture**

Mouse ESC mESC-R1 (ATCC) and mESC-A3 (UC, Merced [[42](#_ENREF_42)]) were maintained on 0.5% gelatin-coated plates in chemically-defined medium containing Knockout Dulbecco’s Modified Eagle Medium (KO-DMEM; Invitrogen), 15% Knockout Serum Replacer (KSR; Invitrogen), 1Χ penicillin-streptomycin (Invitrogen), 1Χ non-essential amino acids (Invitrogen), 2mM L-glutamine (Invitrogen), 0.1mM 2-mercaptoethanol (Calbiochem), 2000 units/ml of leukemia inhibitory factor (LIF-ESGRO; Chemicon), and 10 ng/ml of bone morphogenetic protein-4 (BMP-4; R&D Systems). A3-ESC were maintained in the same media, but co-cultured on 20,000 mouse embryonic fibroblasts (MEF) per cm^2^. Full medium changes occurred every other day and cells were passaged every 4-5 days. Prior to induction, the MEF were purified by gravity separation followed by adhesion to tissue culture dishes for 1-2 hours.

**Human ESC Culture**

Human ESC hESC-H7 (WA07, WiCell) and hESC-H9 (WA09, WiCell) human ESC cultures were maintained with mitomyocin C-treated MEF plated at 20,000 MEF/cm^2^ on 0.5% gelatin-coated plates for 1 day prior to seeding with the human ESC. The human ESC colonies were routinely passaged every 7 days at a ratio of 1:3. The serum-free maintenance medium for the human ESC consisted of: Knockout Dulbecco’s Modified Eagle Medium (KO-DMEM; Invitrogen), 1x penicillin-streptomycin from 10,000 unit stock (Invitrogen), 2nM L-glutamine, 15% KSR, 1x non-essential amino acids, 0.1mM 2-mercaptoethanol, and 5 ng/mL bFGF with a full media change every day.

**Human iPS Cell Culture:** Human induced pluripotent stem cells (DF19-9-7T, WiCell) were maintained on Matrigel hESC-Qualified Matrix (Corning) coated-100mm^2^ dishes in mTeSR^TM^-1 (Stem Cell Technologies) and passed using Accutase (Innovate Cell Technologies) every 4 days.

**Generation of Insoluble VEGF in FN**

FN matrix containing VEGF (VEGF-FN) was generated by mixing 50 μg/mL of FN (BD Biosciences, Corning) in PBS with 5-, 10- or 20ng/mL of VEGF-165 (Peprotech). The solution was allowed to incubate at 37°C in tissue culture well-plates overnight. The following day, immunofluorescence was performed or cell seeding to allow optimal binding time and protein adsorption onto the TC-treated plastic. The percent (%) of VEGF-occupied binding sites, **obs**, was estimated by calculating the number of VEGF molecules, **# VEGF**, added to the total FN, **# FN**, and dividing by the number of VEGF binding sites, **FN bs**, present within the FN matrix.

**VEGF-FN Dissociation**

We assume that VEGF remains bound in the FN. In order to verify that bound VEGF was not being released into the medium, we quantified VEGF-165 release over the 3 days in to PBS using an ELISA kit (Invitrogen) according to the manufacturer’s instructions. The starting concentration of VEGF-165 (20 ng/mL) was diluted to an appropriate amount (750 pg/mL) for the kit’s recommended standard curve (0 pg/mL – 1500 pg/mL). Aspirates of PBS on VEGF-FN were tested for soluble VEGF over 3 days.

**Insoluble VEGF-FN Verification**

Insoluble VEGF-FN blends were plated in a 12-well tissue culture plate (Corning, Costar^®^) overnight before being stained with a primary rabbit anti-fibronectin antibody (Abcam) and a goat anti-VEGF antibody (PeproTech). Both antibodies were used at a dilution of 1:200 in PBS and incubated for one hour each before being rinsed. Secondary anti-rabbit PE (Santa Cruz) and anti-goat FITC (Santa Cruz) antibodies were used to counter stain the primary antibody. Again, both antibodies were used at a dilution of 1:200 in PBS and incubated for one hour each before being rinsed. In order to facilitate imaging at the surface, a glass Pasteur pipette (Fisher) was used to scratch the surface of the plate. Imaging was performed on a Nikon TE2000.
