# Supplementary figures and images for: Multifactorial Optimizations for Directing Endothelial Fate from Stem Cells
Source: PLoS One. 2016 Dec 1;11(12):e0166663. doi: 10.1371/journal.pone.0166663 (PMC5131944; doi:10.1371/journal.pone.0166663)

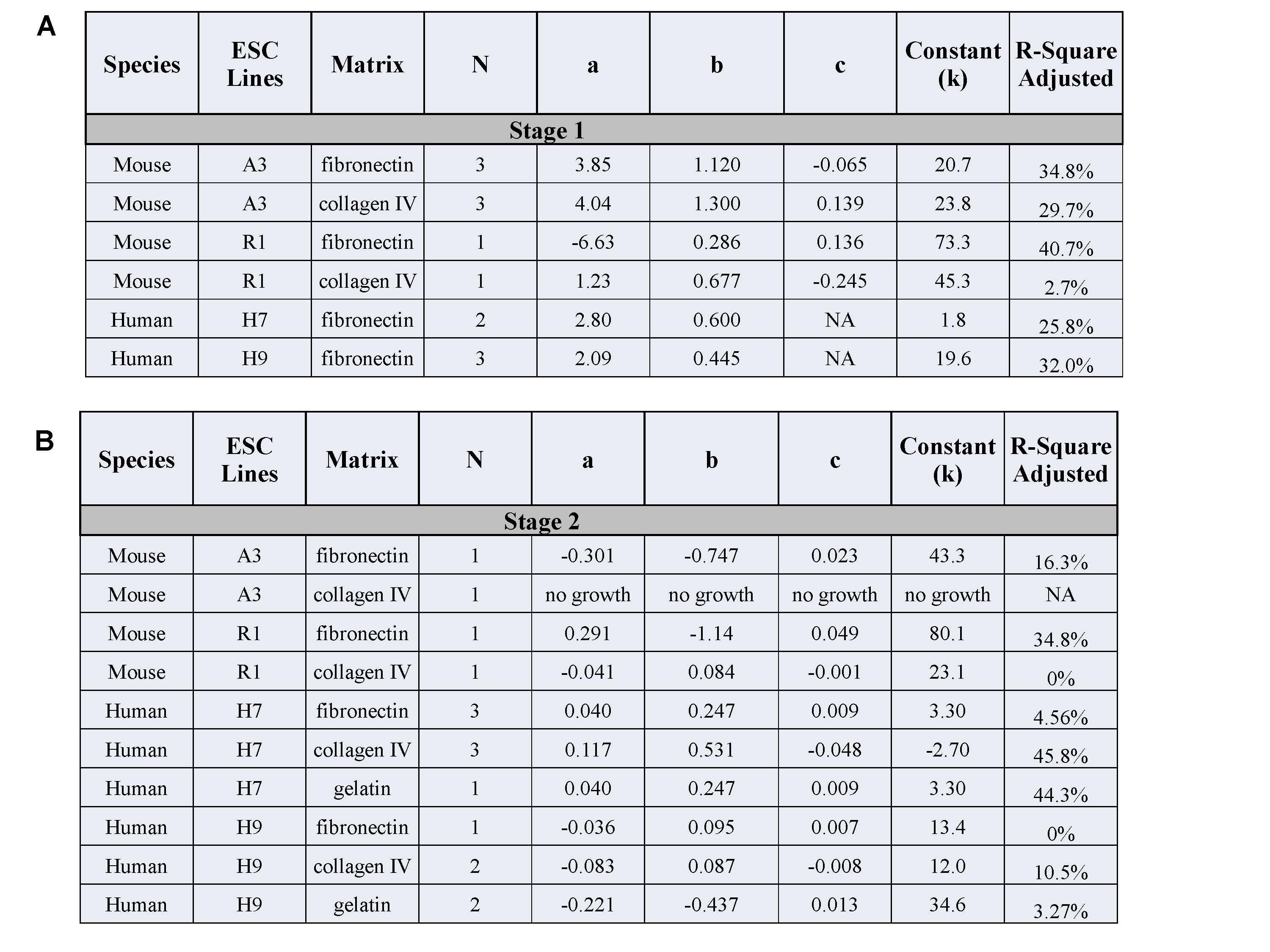

Supplement: S1 Fig — Constants from multiple linear regression models for induction of A) ESC into VPC and B) VPC into VE-cad+ EC. Example of C) trace plots of coefficients fit by lasso and D) cross-validated MSE of lasso fits. (TIFF) [file pone.0166663.s002.tiff]

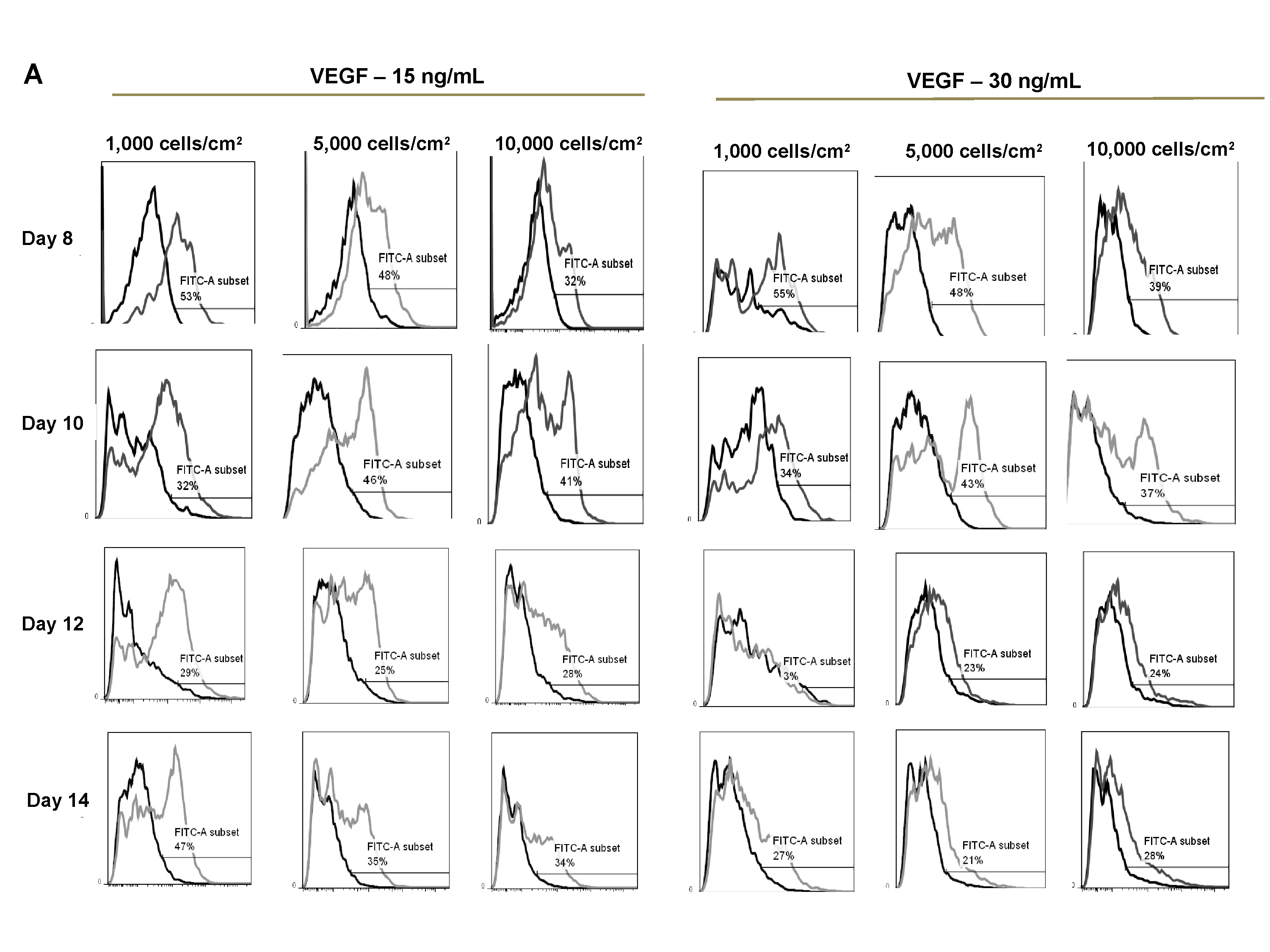

Supplement: S2 Fig — Human iPS cells were examined for A) optimal induction time, seeding density, and VEGF treatment for stage 1 in an iPS cell line. The greatest percentage of KDR+ cells is obtained at day 8 from cells seeding at 1,000 cells/cm2, with VEGF treatment between 15 and 30 ng/ml insignificant. The incorporation of a Wnt agonist (GSK3β inhibitor, called CHIRR99021) was examined from day 0–4 in B) human iPS cells and C) H9-ESC, but did not lead to an increase in KDR+ cells at any time point. (TIFF) [file pone.0166663.s003.tiff]
